# Blueprint for building a biorepository in a resource-limited setting that follows international best practices

**DOI:** 10.4102/ajlm.v8i1.722

**Published:** 2019-08-28

**Authors:** Alash’le G. Abimiku, Talishea Croxton, Petronilla J. Ozumba, Ndidi Agala, Olasinbo Balogun, Emmanuel Jonathan, Enzenwa Onyemata, Kachimi Ndifon, Sunji Nadoma, Thankgod Anazodo, Sam Peters, Christine M. Beiswanger

**Affiliations:** 1 Institute of Human Virology Nigeria, Abuja, Nigeria; 2 Institute of Human Virology, University of Maryland, Baltimore, Maryland, United States; 3 Coriell Institute for Medical Research, Camden, New Jersey, United States; 4 Independent contractor, Philadelphia, Pennsylvania, United States

**Keywords:** biorepository, biobank, international guidelines, best practices, developing country

## Abstract

**Background:**

Genetic diversity is abundant on the African continent. However, genomic research has been hampered by a lack of high quality and extensively annotated biospecimens and the necessary infrastructure to support such a technology-intensive effort.

**Objective:**

The Institute of Human Virology Nigeria (IHVN) partnered with the H3Africa Consortium and the Coriell Institute for Medical Research to build an internationally recognised biorepository for the receipt, processing, storage and distribution of biospecimens for biomedical research. Here, the authors describe the procedures, challenges and results encountered.

**Results:**

Key requirements for a high-quality biorepository were identified: (1) institutional support of infrastructure and services, (2) on-site trained staff with primary commitment to the biorepository, (3) reliance on best practices from globally recognised biorepository groups, (4) early implementation of a quality management system, (5) adoption of a laboratory information management system with demonstrated versatility in functions, (6) collaboration with external experts and sharing of experience through abstracts, newsletters, published manuscripts, and attendance at meetings and workshops, (7) strict adherence to local and national ethical standards and (8) a sustainability plan that is reviewed and updated annually.

**Conclusion:**

Utilising published best practices of globally recognised experts in the biorepository field as a benchmark, IHVN expanded and reorganised its existing laboratory facility and staff to take on this new purpose.

## Introduction

Although the African continent is rich in genetic diversity that informs the search for determinants of disease, genomic research in Africa has lagged due to shortfalls in trained scientists and the necessary infrastructure. The hallmark of genomic research in more developed regions has been high-capacity computing networks and the existence of large collections of well-annotated biospecimens of exceptional quality and integrity in biorepositories that strictly adhere to best practices prescribed by leaders in biorepository science such as the National Cancer Institute, United States,^1^ the International Society for Biological and Environmental Repositories (ISBER)^2^ and the Public Population Project in Genomics and Society (P³G).^3^

In 2010, a partnership between the National Institutes of Health (NIH; United States) and the Wellcome Trust (United Kingdom) fostered the development of the Human Heredity and Health in Africa (H3Africa) initiative for genomic research to be led by African scientists. It would involve the training of young African scientists and the establishment of resources to study the interplay of complex genetics and environment in disease susceptibility, progression, and treatment.^4^ One unique and important component of the H3Africa Consortium is the establishment of regional biorepositories for the collection, quality control (QC), and future distribution of samples and associated data to researchers, using best practices, as new insights into genomic medicine are gained. The three regional H3Africa biorepositories are: the Institute of Human Virology Nigeria (IHVN) H3Africa Biorepository (I-HAB) in Abuja, Nigeria, serving western Africa; Clinical Laboratory Services in Johannesburg, South Africa, serving southern Africa; and the Integrated Biorepository of H3Africa Uganda (IBRH3AU) in Kampala, Uganda, serving eastern Africa.^5,6,7,8,9,10,11^

The IHVN, the largest non-governmental organisation in Nigeria providing HIV and tuberculosis prevention, care and treatment programmes under the US government President’s Emergency Plan for AIDS Relief programme and the Global Fund partnered with the Coriell Institute for Medical Research, Camden, New Jersey, United States, in 2012 to achieve international biorepository recognition by adopting the best practices, in particular those of the ISBER.^1,2,3^ This was to be accomplished through a combination of staff training, auditing by an independent expert consultant, renovation and acquisition of additional dedicated storage space, and upgrading of critical equipment and information systems.

The principal goal of I-HAB is to provide reliable sample processing support, secure storage and shipping of high quality biological samples. Here we report on the processes, successes and challenges of establishing such a biorepository in the context of a resource-limited setting. The knowledge gained from this undertaking can inform the establishment of laboratory facilities serving a variety of clinical and research purposes in low- and middle-income countries, with a committed staff, creative problem-solving and careful planning as we have shown.

## Strategy and planning for an International Society for Biological and Environmental Repositories compliant biorepository

### Ethical considerations

Ethical clearance was obtained from the National Health Research Ethics Committee, approval number (NHREC)/Biob/01/02/2018 (22 March 2018–22 March 2020).

### Institutional support for staffing and infrastructure

To establish the new biorepository, renamed I-HAB, a professor-level laboratory director, management staff and three technical staff were reassigned from the IHVN to I-HAB. In addition to funding the initial renovation and expansion of the existing laboratory and storage facilities based on the audit reports, IHVN committed resources and staffing for maintaining and securing the facility, and for information technology support.

### Readiness assessment

**FIGURE 1:**
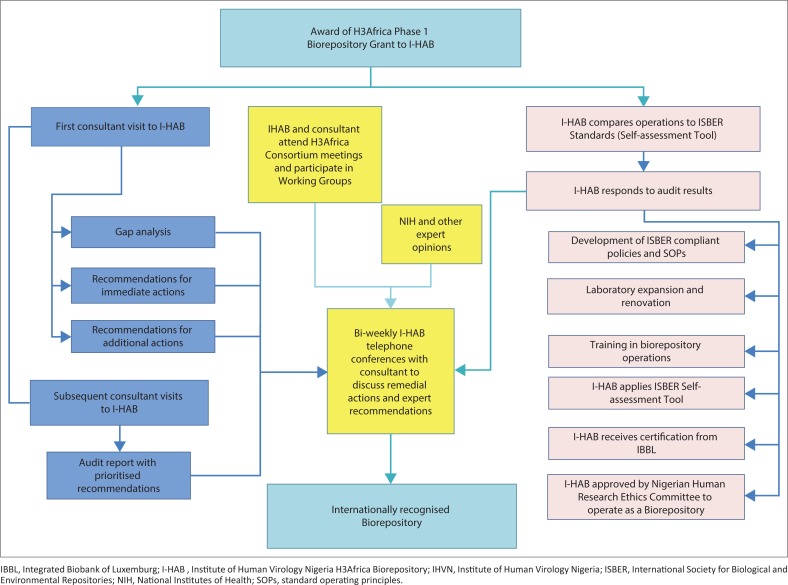
Flowchart of the process to bring IHVN H3Africa biorepository to international biorepository standards. Activities undertaken by the Institute of Human Virology Nigeria-H3Africa Biorepository to achieve compliance with Biorepository ‘Best Practices’ are shown in coloured boxes: dark aqua are initial activities and final outcomes; pink are activities undertaken by IHVN H3Africa biorepository staff; medium blue are activities carried out by the independent consultant; yellow are major policy determinations.

The I-HAB conducted a number of baseline assessments of its readiness to operate according to international biorepository practices using the ISBER self-assessment tool.^5^ The ISBER checklist evaluated the biorepository’s records, management reviews, organisation and personnel, client management, equipment, internal audit, purchasing and inventory, information management, process control and external quality assurance, corrective actions, occurrence and incident management, and facilities and safety (Figure 1). The assessment analysis received from ISBER was incorporated into a remediation plan and the categorised outcomes and overall score were used to monitor progress.

Additionally, the World Health Organization Stepwise Laboratory Quality Improvement Process Towards Accreditation (SLIPTA) checklist^6^ was used to ensure compliance with ISO 15189 standards suitable for I-HAB’s added role in supporting clinical sites. The SLIPTA checklist assessed management, security, accuracy, record keeping, and standard operating principles (SOPs) but from a slightly different perspective that augments the ISBER self-assessment tool.

### Independent biorepository consultant

In partnership with the consultant from Coriell, I-HAB proposed a strategy of cyclic assessments and remediation to identify areas of improvement and to bridge identified gaps. During several trips to I-HAB, the consultant worked with I-HAB to identify disparities between existing IHVN practices and the widely accepted practices of biorepositories serving international studies. Once the gaps were identified, plans for improvement were drawn up and implemented. Figure 1 captures the overall process to upgrade the practices at I-HAB using an audit tool developed by the consultant as detailed in Table 1. The audit tool addressed areas of operation important to the mandate of the H3Africa biorepositories. For example, QC of DNA was included, but not processing of formalin-fixed tissue samples.

**TABLE 1:** Audits by the independent consultant show incremental improvement.

Date	Assessment type	Score (%)
October 2012	Initial baseline	70.0
May 2013	Audit and training	81.9
May 2015	Second audit and training – Pre-NIH visit audit	93.6

NIH, National Institutes of Health.

**FIGURE 2:**
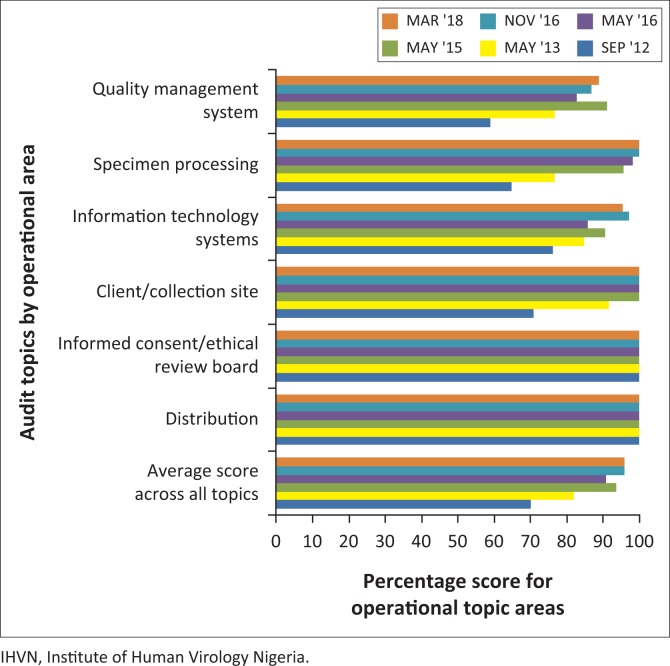
Audit topics and results provided by IHVN H3Africa biorepository consultant. This graph presents a quantitative assessment of IHVN H3Africa biorepository performance on each external audit by the independent consultant. Horizontal axis: proportion of the maximum possible score. Vertical axis: major operational areas examined during each audit.

Operations requiring immediate remediation received a score of 3; necessary, but not critical, improvements received a score of 2; strongly suggested improvements scored 1 and acceptable ‘as is’ practices received a score of 0. Scores were totalled in each operational area and subtracted from the possible number of points in that area and a percentage of the possible score calculated (Figure 2). The full report was used as a reference to address non-conformities prior to the next audit site visit, and to track improved compliance with ISBER best practices over time.

### Adoption of a quality management system

The I-HAB already followed the principles of a quality management system (QMS) in line with its role as the biorepository for other IHVN research studies. However, the following critical quality components were enhanced.

#### External quality assurance scheme

Enrolment in the Integrated Biobank of Luxemburg (IBBL)^7^ external quality assurance scheme meant DNA extraction, quantification, and purity assessment from whole blood was performed annually. After verification of results, IBBL issues certification of proficiency. So far, I-HAB has received a certificate of satisfactory performance from IBBL for all four annual reports since 2015.

#### Continuous quality improvement

Regularly scheduled meetings for review of all operations and planning activities are fundamental to reach and sustain quality. I-HAB has three categories of review meetings. First is the weekly meeting of all I-HAB staff, second is a bi-weekly meeting with the director, technical advisors and IHVN top management, and third is an alternating bi-weekly meeting with other regional H3Africa biorepositories when the sponsors participate. In addition, I-HAB staff attended the bi-annual H3Africa Consortium meetings to ensure an appreciation of the broader consortium’s opportunities and challenges, to learn from each other’s experiences and to develop consortium-wide strategies. Collaborations with other H3Africa investigators^9,10^ facilitated I-HAB’s internal review and mastery of unique biobanking needs.

### Training/mentoring

Training in biorepository principles and practices was provided to different categories of staff as necessary: the I-HAB management staff, technical staff, support staff from other IHVN departments, and research staff at H3Africa-supported clinical sites. Training consisted of didactic presentations and discussions of biorepository policies and practices, interactive development of cost of service schedules, and wet labs. All training materials (presentation slides, review versions of SOPs, electronic links to background materials) were made available to participants for future use.

### Implementation of a biospecimen inventory and laboratory information management system

The I-HAB adopted Freezerworks (©2015, Dataworks Development Inc., Mountlake Terrace, Washington, United States) as a biospecimen inventory system. Freezerworks is a commercially available laboratory information management system (LIMS) with good technical support, user-defined fields and reports, easy modifiability and frequent upgrades by the company to remain current with new technologies and features of a biorepository LIMS. It is able to track QC and distribution of samples as well as in-house inventory. Additionally, Freezerworks is compatible with most data sharing protocols and automated data capture for laboratory equipment.

### Ethics and governance of banking of biological samples

The I-HAB serves as the ‘custodian’ of biospecimens stored at its facilities. The I-HAB can return biospecimens to the submitter, redistribute or share them, use them for research, or dispose of them only upon authorisation by the original submitter of the biospecimens or an authorised agent. The governance of the H3Africa biospecimens deposited with I-HAB is guided by approved informed consents for the various studies and the policies of the H3Africa Consortium.

### Sustainability planning and costing of biorepository services

The NIH funding supporting the H3Africa biorepository programme will eventually cease and so planning for continuation of I-HAB is crucial. Sustainability of I-HAB will require a multipronged approach with excellent revenue tracking and careful management of partnerships with commercial enterprise. The I-HAB developed a costing model to determine the actual costs of its services. This tool can be used for a fee-for-service scheme ensuring that pricing does not fall short of covering its operational costs. For each service, a direct cost based on labour plus reagents and supplies and pro-rated cost of instrumentation was calculated. Labour included technical and management time at a ratio of 90:10. Services were costed by individual sample or by a minimum batch size. An ‘overhead cost’ as a percentage of the direct cost and a mark-up cost that would cover contingencies and future service development were added. This mark-up was considered to be negotiable for I-HAB to remain competitive and depending on the potential customer’s resources or opportunity for collaborative efforts.

## Outcomes of implementation measures

### Staffing and management organisation

**FIGURE 3:**
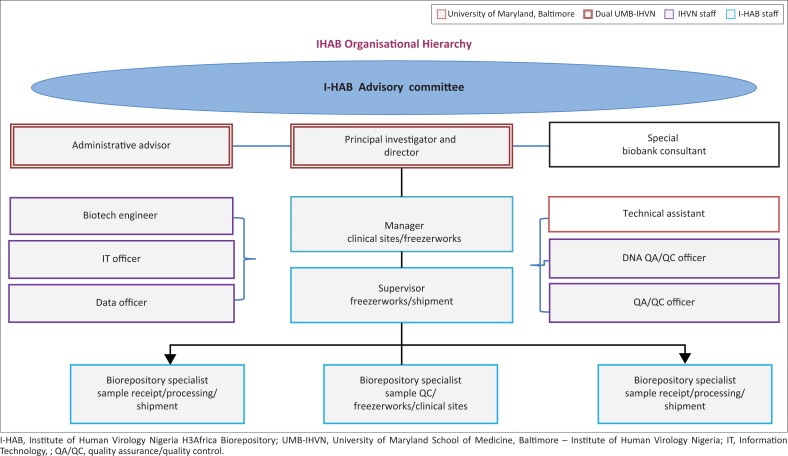
IHVN H3Africa Biorepository organisational chart and staff responsibilities. The IHVN staff positions shared with IHVN H3Africa biorepository are indicated by the purple outline; solely IHVN H3Africa biorepository staff by the aqua outline; University of Maryland, Baltimore by the red outline; dual IHVN/UMB by the double red outline; technical advisor and consultant by white boxes.

The I-HAB is guided and directed by a director who benefits from input from an advisory committee and technical experts to set goals in accordance with the I-HAB and IHVN missions, the H3Africa objectives and client requirements, as well as to develop and strengthen policies and procedures, and provide mentorship. A biorepository manager has the primary responsibility of organisational management, overseeing the QMS and safety, ensuring activities are conducted ethically, contributing to the development and maintenance of a sustainability model, and training. Two scientists with expertise in molecular biology were employed in light of the anticipated increase in biospecimen processing and QC associated with supporting H3Africa projects. These positions as well as others are shown in I-HAB’s organisational chart (Figure 3). All staff members are cross-trained and involved in the overall QMS activities, in addition to attending internal and external meetings, presenting at workshops and trainings, contributing to document creation and review, preparing manuscripts, and ensuring security of the facility, biospecimens and data.

### Biorepository infrastructure and security

#### Infrastructure

**FIGURE 4:**
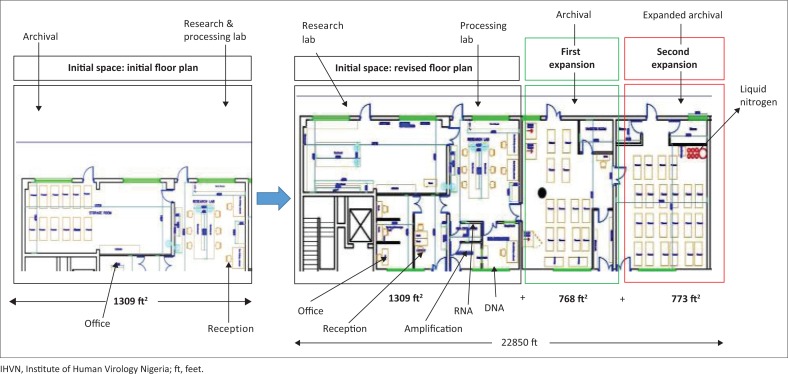
Expansion of IHVN H3Africa biorepository infrastructure to support an international biorepository. The initial 1309 ft^2^ biorepository space is shown in the black outline of the left of the figure. This space was reconfigured and a 768 ft^2^ cryogenic room was added (green outline). A second expansion (red outline) added 773 ft^2^ of secure biorepository storage space.

The 1309 ft^2^ I-HAB facility shown in the black boxes in Figure 4 was expanded to 2077 ft^2^ to improve workflow and separation of processes, in anticipation of 100 000 DNA samples to be processed and stored each year for H3Africa projects in West Africa. The green and red boxes in Figure 4 depict the completed two expansions. Major re-organisation created the following laboratory spaces: (1) a general biospecimen processing laboratory, (2) archival rooms, (3) DNA processing room, (4) RNA processing room, and (5) amplification room. Facility upgrades include: restructuring of the manager’s office, installing epoxy flooring to prevent spills from being trapped and to enable floors to be sanitised, installing additional power and internet outlets to support more equipment and prevent usage of extension cords, elevating electrical outlets from the base of the floor as a safety precaution, installing a generator with a second one as backup to supplement the public power supply, and installing two inverters and numerous uninterruptible power supply devices to ensure constant power and to minimise power fluctuations. The two expansion spaces accommodate nine ultra-low temperature freezers, two refrigerators, three liquid nitrogen tanks, and one auto-fill liquid nitrogen tank. Overall, I-HAB utilises about 7 983 360 watts of power each week. Only 58.3% of the necessary power is provided by the public power grid; the rest (41.7%; 70 hours per week) is provided by backup generators with an automatic switch during local power grid outages. The backup to ensure 24/7 power has been utilised for a total of 20 993 hours since 2012, when I-HAB began its journey to achieve international best practices, until early 2016, when this manuscript was put together. The power generators are tested at full capacity for 72 hours once a year to ensure uninterrupted service in case of prolonged power outage. Furthermore, backup plans include supply of liquid nitrogen or off-site storage of critical biospecimens in case of prolonged power outage.

#### Security

**FIGURE 5:**
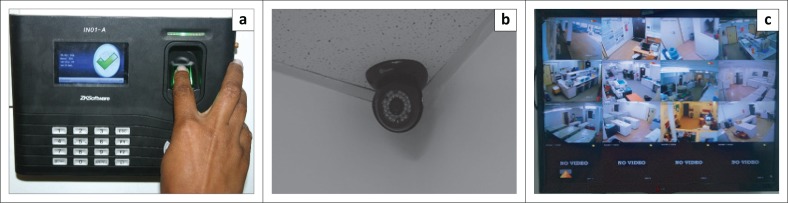
Security installations at Institute of Human Virology Nigeria H3Africa biorepository. (a) Biometric access control panel. (b) Motion-activated closed circuit television camera. (c) Monitor display from closed circuit television camera showing both interior and exterior areas of biorepository.

Written policies, SOPs, and upgrades were implemented to ensure that there are processes and procedures established that minimise risk for security breaches and dictate response in case of emergency. Biometric devices (ZKTeco, Tangxia Town, Dongguan, China) and closed circuit television cameras (Swann Communications, Santa Fe Springs, California, United States) and monitors were installed to prevent unauthorised access and improve staff, biospecimen and data safety and security (Figure 5). Automatic data backups were initiated both to off-site servers and on-site external drives to prevent data loss. The I-HAB provided formal training in emergency response to all IHVN security staff charged with 24-hour security of I-HAB.

### Self-assessment results

The I-HAB scored 66.9% at baseline in December 2012 using the ISBER self-assessment tool (Table 2). Comments returned from ISBER are described in column 2 of Table 2 and issues identified were addressed with mentorship from the consultant for a second ISBER self-assessment conducted in January 2014, when I-HAB scored 88.2%. Following remedial action to again address the issues raised, a final ISBER self-assessment conducted in July 2015 had an improved score of 95.0% (Table 2). This underscores the need for continuous self-assessment and improvement to ensure quality and adherence to international best practices especially in light of newly added activities or processes.

**TABLE 2:** International Society for Biological and Environmental Repositories self-assessments show incremental improvement from 2012 to 2015.

Date	Findings	Score (%)
December 2012	Lack of physical monitoring of the facility from off-site Need for a standardised process for regular backup of electronic records No provisions to ensure the good working order of electrical cords, plugs and power outlets	66.9
January 2014	Lack of physical monitoring of the facility from off-site was still not addressed satisfactorily Documentation of time between collection of specimen and processing or preservation Use of specimen stabilising agents	88.2
July 2015	Need to characterise specimens for identification purposes Need to maintain records for equipment purchases Need to document electronic inventory system validation	95.0

An assessment was also performed by IHVN laboratory quality assurance officers in February 2013 using the World Health Organization SLIPTA checklist.^6^ While the score of 76.3% (Table 3) is not directly comparable to the ISBER self-assessment tool score, it does suggest improvement in the management and operational activities of I-HAB over the previous year’s score of 66.9%.

**TABLE 3:** World Health Organization laboratory self-assessment.

Date	Findings	Score (%)
February 2013	Need for Hepatitis B virus vaccination for all staff Exit doors should not lead to open drainage Fabric covered chairs should not be used as they cannot be decontaminated Electrical sockets for freezers should not be mounted directly on the floor Personnel files lacked Competency Assessments No document archival system Effective dates and annual review dates not captured on standard operating procedures Acceptance and rejection criteria of consumables not filed so that they are easily available Some equipment not calibrated Inadequate temperature monitoring	76.3

### Audits of policy and practices by independent consultant

Beginning in October 2012, the Coriell consultant implemented a schedule of rigorous formal audits of biorepository activities during three on-site visits. Each audit was followed by a detailed report, which the staff used to address gaps identified during the audit (Table 4). The initial assessment by the independent consultant indicated that I-HAB exhibited reasonably good compliance (70%) with the best practices recommended by ISBER and NIH in the areas of management, specimen processing, information technology systems, client and collection site interactions, informed consent and ethical reviews, and policies for distribution of biospecimens (Figure 2). Each subsequent audit exhibited an improvement in scores (81.9% in May 2013 and 93.6% by May 2015), suggesting that the remedial activities and formal biorepository training were effective tools for improving biorepository operations (Table 1). The ethics and distribution areas were in total compliance from the onset of the H3Africa Phase I, because I-HAB had been operating in these areas in earlier contracts from United States government funded grants^11,12^ where I-HAB followed all the ethical requirement of the contracts.

**TABLE 4:** Major deficiencies during formal audits.

Operational area	Initial finding	Status as of May 2016
QMS	Missing or inadequate SOPs	Mostly complete; 159 documents created and adopted
	Staff organisational charts not clear	Dedicated staffing in place
	Need more documentation of management activities	Additional SOPs have been developed
	Lack of training programmes for support staff (sample drivers, physical plant)	Programmes are in place with appropriate SOPs and documentation of trainings
	Some SOPs are missing or require revision	Over 160 documents revised/created
	Need better SOP and training for backup generator operation	Training course has been implemented
	Need documentation of training for generator operation	Training course has been implemented and only approved security staff have access to I-HAB
	Need more ‘management’ SOPs	Newly discovered SOPs needed and have been created as operations become more sophisticated
	Inadequate layout of physical space	Extensive renovation and acquisition of additional space; layout of existing facility redesigned to maximise operational effectiveness and air flow
Specimen processing: infrastructure	Lack of remote temperature monitoring	Mostly complete; web-based monitoring and email alerts active, text alerts in progress; 24-hour human coverage by security personnel, audible alarms
	LIMS (Freezerworks) procedures need to be more rigorous	Additional training and installation will facilitate achieving this goal
	Additional physical security measures are needed (controlled access to I-HAB, intrusion alarms, a 24/7 response protocol)	Biometric entry system, surveillance camera and monitoring system, 24-hour security, 24/7 response protocol installed and only approved security staff have access to I-HAB
	Power disruptions require the use of UPS on all critical equipment	All critical equipment have UPS
	Lack of remote temperature monitoring	SmartVue remote alert and monitoring; testing of a second system (Tutela temperature monitoring equipment) as backup
	Insufficient biospecimen backup storage at operating temperature and alarmed	More than 10% backup as recommended by ISBER best practices in place
	Remote temperature monitoring system has been installed, but notification is not fully functional	Email notifications ongoing, text notification in progress; working with manufacturer to resolve
	Remote monitoring of temperature of storage units and a plan for response and documentation of events	SmartVue and Tutela remote alert and monitoring, SOP for emergency response developed and practised
Informed consent/ethical review boards	Contact information and MTAs and informed consent need to be documented for each study	Acquired for all studies supported by the biorepository
Distribution	Need to develop additional records of sample retrieval and distribution	Additional training and planned installation of Freezerworks upgrades will facilitate achieving this goal
	Need IATA training for all staff shipping samples	All staff have been trained on IATA shipping procedures
Information technology system	No systematic backup of LIMS system	Routine automatic back up to off-site server and on-site external drive in place
	No provision for automatic backup of LIMS	Routine automatic back up to off-site server and on-site external drive in place
	Need to re-enter data in LIMS that was lost during a backup procedure	Procedures have been instituted to prevent a reoccurrence
	Need to restore LIMS from backup copy	Restoration procedure conducted routinely
	Automatic backup of the LIMS exists, but restoration from a backup copy has not been tested	Backup restoration tested and routinely scheduled

FW SOAP, firewall simple object access protocol; IATA, International Air Transport Association; ISBER, International Society for Biological and Environmental Repositories; LIMS, laboratory information management system; MTA, Material Transfer Agreement; SOPs, standard operating procedures; QMS, Quality Management System; UPS, uninterruptible power supply.

In summary, over the course of two and a half years, I-HAB had demonstrated consistently improving operation as a biorepository in achieving international best practices. A summary of the major deficiencies found during the independent consultant audits is captured in Table 4. There were 14 significant non-conformances at the first audit in October 2012. Half of these were resolved by the second audit in May of 2013 and only two critical gaps remained by the third audit in May of 2015. Both issues, automatic notification of staff regarding temperature conditions in biospecimen storage units, and a plan to regularly document perfect restoration of the biospecimen database from a backup copy, relied on external suppliers.

### Changes/enhancements to quality management system

#### General biorepository operations

Shipping policies, practices, forms and SOPs were written as generic documents for I-HAB and the H3Africa Consortium before being customised for specific projects. Included in the documents prepared to assist submitters in the deposition of biospecimens are: (1) SOPs on sample preparation, labelling, shipping conditions, form preparation, and communication with the biorepository, (2) material transfer agreement templates that can be adjusted to meet the regulatory requirements of the submitter’s country, (3) pre-shipment checklists to ensure that all supplies and forms are available and that all arrangements have been established with the biorepository and with the chosen shipping courier, (4) a shipment manifest form indicating the sample type, sample identification number and accompanying minimum sample essential data, (5) a shipment notification form to inform the biorepository of the shipment date and other arrangements, and (6) a shipment receipt confirmation and query form used to address any discrepancies in the shipment.

In addition, more than 150 policy documents, SOPs and forms were created or revised in compliance with best practices and in response to site assessments and client needs. All documents include information on unique identification, versioning, review and sign-off by staff, and are in a document inventory for tracking. To more effectively update and review newly created and revised documents, meetings were held where authors presented the document to staff in an interactive session. Questions and other feedback were addressed before adoption of documents.

#### Staff training and continuing education

Formal training sessions were provided to all I-HAB staff as detailed below and in Table 5. Pre- and post-training assessment quizzes were used to determine the success of the training. Staff were also re-evaluated annually. Training included:

**TABLE 5:** Summary of I-HAB and IHVN support staff training of IHVN H3Africa biorepository staff training.

Trainings pre-H3A	No. trainees	Training post-H3A	No. trainees	Training post-H3A	No. trainees
Basic laboratory trainings	6	Modern Research Bioethics	2	Beta-testing for Freezerworks	1
IATA certification	4	Freezerworks training in San Diego	2	SPREC orientation	5
Freezerworks	8	Bioinformatics course in Kenya	1	Safety	5
	-	Grant management training; NIH Bethesda	1	DNA quantification by Qubit	3
	-	Biorep training (includes IATA Regulations)	8	Sample collection PAXgene and Oragene	3
	-	Biorep management training	10	DNA extraction from oral fluid	3
	-	Biosafety training for drivers and cleaners	30	DNA extraction PAXgene kit	3
	-	CITI online course	4	Safety training for I-HAB cleaner	1
	-	Biohazard waste management and waste disposal Biorep Staff	10	Emergency response and safety training for I-HAB security and maintenance officers	9
	-	Basic Biorepository Training (for H3A Kidney Disease Research Network sites in Nigeria)	12	MagNA Pure and Light Cycler	5
	-	Emergency Response	6	Freezerworks New Customisation	7

CITI, collaborative institut-ional training initiative.IATA, International Air Transport Association; IHVN, Institute of Human Virology Nigeria; NIH, National Institutes of Health; SPREC, sample preanalytical code.

**Basic laboratory operations:** Good laboratory practices and workflow, specimen management, quality management, quality assurance, quality control, quality assessments, continuous quality improvement, SOP writing, and safety.

**Introduction to biobanking:** Biospecimen receipt, processing, aliquoting, labelling, storage, retrieval, transport, DNA extraction, quality control methods, International Air Transport Association regulations, data management, documentation and record keeping.

**Biorepository management workshop:** Expanded training on some of the above topics, including accreditation processes, biorepository best practices, biorepository services, cost modelling for services, disaster planning, critical samples, stakeholders, funding and establishing policies.

**Modern research bioethics:** History of research ethics and research regulation; legal, moral and philosophical foundations of modern bioethics; informed consent, exploitation; benefits, inducements and compensation for research injuries and vulnerable populations; ethics committees; types of ethical review; scientific integrity and research misconduct; evolution of and process for ethical review in Nigeria.

**Bioinformatics course:** Attended by the Information Technology officer, this course included Linux operating systems, Python programming, advanced dataset management with Linux, population and statistical genetics and next generation sequence analysis.

**Grants manager course:** Participation in a three-week residency training component of Biomedical Research Administration Development at NIH. The Biomedical Research Administration Development programme is designed to: help participants to navigate and understand the NIH structure and programme operations, introduce participants to NIH grant policy and compliance requirements, and provide an overview of the knowledge base and tools for building a strong research administration support and management infrastructure at the participant’s institution.

**Freezerworks:** The I-HAB’s bioinformatics specialist, laboratory manager, and technical advisor attended Freezerworks update training sessions provided by the manufacturer. Step-down training was provided to all I-HAB technical staff who were invited by Dataworks as beta tester for Freezerworks 2015. This experience provided an excellent opportunity for the staff to suggest feedback to the manufacturer.

### Information technology and laboratory information systems

As mentioned in the Methods section, the I-HAB uses Freezerworks to manage biospecimen and associated data. There have been several upgrades since Freezerworks was initially installed at I-HAB in 2012. The most recent version, Freezerworks 2015 Ascent (©2015, Dataworks Development Inc., Mountlake Terrace, Washington, United States), is a networkable system with web access that enables tracking of samples from biospecimen collection through storage and distribution. All processes carried out on a biospecimen, such as testing, shipping, freeze-thaw cycles and other frequently required biospecimen modifications, were documented. The security feature was used to assign authorities for specific activities like viewing, modifying, adding data, as well as administrative duties such as customisation of the program. All activities are tracked by an audit trail and can be traced to the individual user. Other useful functions used were import, export, and batch update of data, and generation of reports in Excel, Word or PDF.

**FIGURE 6:**
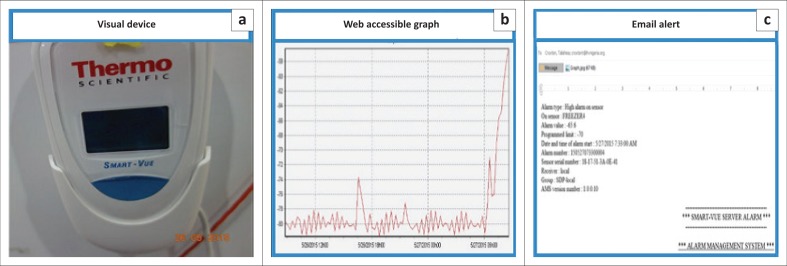
Remote temperature monitoring. Features of the SmartVue Temperature System are shown. (a) Digital temperature display available on individual ultra-low temperature freezers. (b) Continuous temperature readout for each freezer accessible on Web. (c) An email alert is sent whenever the temperature of an individual freezer exceeds limits set by biorepository management.

The I-HAB purchased a temperature monitoring system to permit remote monitoring of all ultra-low temperature freezers and for automated remote alarm notification in case of a temperature excursion beyond preset limits (Figure 6). The SmartVue (Thermo Fisher Scientific, Waltham, Massachusetts, United States) remote temperature monitoring and alert system did not work in Nigeria due to incompatibility with the existing network system operating in the country, but the Tutela (Tutela Monitoring Systems, Hampshire, United Kingdom) worked. The system provided mechanisms for: digital visual readings, web accessible readings and graphs for remote monitoring in real time, and email and text alerts that enable the staff to respond. I-HAB established an on-call schedule and response communication tree to ensure that staff members monitored and responded to emergencies during nights, weekends and holidays as well. In addition, IHVN security guards were trained in appropriate response protocols and have 24-hour access to respond to audible temperature alarms in the I-HAB facility and log their responses.

### Governance and ethical considerations

The National Health Research Ethics Committee (NHREC)^13^ has guided I-HAB in developing practices that are in line with the National Code of Health Research Ethics of Nigeria. Regular interactions with members of NHREC and the H3Africa funded research projects on ethical, legal, and society issues and the guidance documents produced by these groups has enabled I-HAB to carry out its operations ethically according to the regulatory and social norms of Nigeria and the other African countries it serves. The NHREC visited I-HAB to observe and inspect its practices before licensing it to function as the first Nigerian biorepository meeting the special requirements of the functions of a biorepository.

### Sustainability and costing

The I-HAB sustainability plan is a multipronged approach to reduce or contain current costs. Contracts were established with couriers and vendors whenever possible. I-HAB management identified consumables and equipment required for expansion and sustainability and investigated vendors that provided those products and support to Nigeria, those with offices in African countries and others that were well-known international third-party distributors. Contracts for discounted pricing and services were negotiated with Thermo Fisher Scientific and DNA Genotek (DNA Genotek, Ottawa, Ontario, Canada) and discussions are ongoing with others.

The I-HAB negotiated prices and services for transport of biological materials. Couriers were compared for their ability to transport biospecimens between the H3Africa collection and processing sites and the I-HAB. The duration and cost of the shipments as well as the provision of supplies for ambient, refrigerated and frozen shipments were assessed. Due to varied and high costs associated with dry ice supply and replenishment required for some biomaterials, Credo reusable shippers (Pelican Biothermal, LLC, Plymouth, Minnesota, United States), equipped with robust insulation to maintain temperatures of −20 ^o^C to −45 ^o^C for many days were preferred.

The I-HAB also developed a model for pricing all biorepository services that was flexible enough to adapt to both rising costs for labour and supplies and that could respond to individual circumstances of potential clients. The exhaustive list of services offered by I-HAB would permit potential clients to customise their contract to include only the minimal requirements for safe and secure processing and storage, or the high-end quality control and customised reporting. The mark-up percentage was adjusted up or down to reflect potential collaborative efforts or other benefits that I-HAB might realise as a result of the contract.

## Context, successes, challenges and future plans

The advent of genomic medicine has underscored the need for mechanisms to make large cohorts of biospecimens and their associated datasets available to researchers across a variety of biomedical fields.^4,14,15,16^ These ‘data-driven’ research efforts are supported by the thousands of biospecimens stored in biorepositories. However, despite its burden of disease, Africa has lagged in the development of biorepositories to provide high-quality samples for collaborative research across biomedical disciplines. African biorepositories have tended to be focused on single diseases in narrowly defined populations and for short periods of time. Some operate as partners in United States- or European-funded collaborative studies^17,18,19,20^ of specific diseases. Others are part of hospitals or academic centres that have restrictions on the use of biospecimens collected and stored for specific studies or clinical use.^21,22,23,24^

Like most institutions in Africa, IHVN had created a biorepository network to support its funded clinical and research activities related to HIV, tuberculosis, and HIV-associated malignancies in 2009. Training in clinical protocols and biorepository activities was provided through its collaboration mainly with the Institute of Human Virology, University of Maryland School of Medicine, Baltimore, United States.^11,12^ The biorepositories had the ability to extract peripheral blood mononuclear cells, and extract DNA from whole blood, QC the samples, store, and ship some of samples for investigative analysis at Institute of Human Virology, University of Maryland School of Medicine using policies and practices specifically mandated by the individual investigator’s research.

With the advent of the H3Africa and expectation to process, ensure the quality of, provide long-term storage for, and distribute biospecimens for international genomic studies, it became necessary for IHVN to scale up its in-house biorepository operations to achieve international best practices. This upgrade of policies and practices ensures that biospecimens passing through I-HAB meet the stringent needs of international biomedical research. This is essential to fulfil I-HAB’s mission to promote population and personal health, by facilitating cutting-edge research and collaborations among African communities and globally, through the provision of high-quality, affordable biobanking services, while maintaining the integrity and confidentiality of these samples as an honest custodian.

Achieving international expertise in biobanking required a substantial revision and the development of a more complex and broader appreciation of biorepository operations. In terms of both self- and external assessments, I-HAB’s performance increased significantly by around 23% from 2012 to 2015 due to improvements including but not limited to staffing, infrastructure, process, and security. The process of achieving international best practices revealed a number of contributing strengths and some serious challenges. However, to obtain such success it was important that the senior management of IHVN and I-HAB were fully committed and willing to obtain the necessary resources to support the development and growth of the biorepository that will outlive H3Africa. This prompt engagement of management staff is a major contributing factor to the success of I-HAB and was the hallmark for sustainability. For example, IHVN provided financial resources for the acquisition and remodelling of the laboratory and cryogenics facilities, information technology services, security systems and staffing, training for I-HAB staff in clinical laboratory practice and other infrastructure operations. Likewise, I-HAB strengthened its own expertise in biorepository operations by offering training courses to other IHVN staff and staff at clinical sites supported by IHVN and other investigators, thus creating a cycle of capacity building and sustainability.

It is important to note that many of the laboratory skills and practices necessary for the operation of a clinical laboratory are also essential biorepository activities. I-HAB had the advantage of a thorough grounding in quality management systems, safety protocols, and equipment maintenance and validation through PEPFAR. Hence the appreciation of strict adherence to SOPs and documentation of activities was an advantage, as well as well-developed bench skills and the application of universal precautions for the handling of human biospecimens, which all became very useful when training and mentoring clinical sites who subsequently shipped biological specimens to the biorepository for storage.

The interaction with H3Africa researchers and experts, through the H3Africa Consortium and two other regional H3Africa biorepositories,^25,26,27,28,29,30,31^ has been invaluable in developing international policies and practices now in place at I-HAB. A number of collaborative manuscripts from the H3Africa Biorepository Working Group have highlighted several relevant concepts, including the role of genomics in African health, how biorepositories in Africa directly support African genomics research, the value of piloting biorepository operations across national boundaries and the complex governance of ‘sharing’ biospecimens for secondary research.^28,29,30,31^

Although I-HAB has successfully developed an ISBER-compliant biorepository, there were a number of challenges that it had to overcome to achieve this. Firstly, resource limitations with regard to infrastructure, especially uninterrupted electrical power and internet access, have been especially challenging to the development of I-HAB: power outages eventually shorten the lifespan of freezer compressors and other instrumentation, the cost of fuel to operate the backup generators fluctuates and must be factored into the expenses of running the repository, the interruption of internet access by power failure exerts a substantial toll on both communications and operations, and modern science is becoming increasingly reliant on international web-based conferences and electronic data transfers, which are all dependent on reliable power and internet. Secondly, technical support from international suppliers can be responsive and timely or it can be expensive and slow or even non-existent. Obtaining the necessary technical help takes assertiveness, persistence and consistent follow-up. Thirdly, acquiring the necessary laboratory supplies and instrumentation can also be expensive and experience delays. Materials that are a quick online catalogue order in a developed country often require additional permits or other paperwork for imports and are likely to have additional costs associated with their delivery and installation. Fourthly, compliance with ethical, legal, and social understandings and regulations also presents challenges to biorepository activities. Thus far, I-HAB has followed ethical practices specified by the Nigerian government,^13^ (http://www.nhrec.net/nhrec/NCHRE_Aug%2007.pdf) and its current contractors, NIH as published by the Office for Human Research Protections (available at http://www.hhs.gov/ohrp/humansubjects/index.html), and the H3Africa Consortium (http://www.h3africa.org/consortium/documents). A full discussion of these issues is beyond the scope of this report, but additional insight can be found in other published articles.^32^ Fifthly, I-HAB has experienced major delays (up to 6–9 months) in obtaining ethical and government approval of material transfer agreements and import permits from a minority of partnering countries. On occasion the wording of a document will need to be changed, but in other cases, there is no existing body of law. It is essential that all transfers of biospecimens to and from a biorepository begin with a careful consideration of ethical, legal, and society issue actions and engagement to be taken and ensure the earliest possible application for required permits.

Finally, reliance on external funding support is increasingly uncertain in a climate of increasing demands to cut government spending. Many funders of research infrastructure in Africa (such as H3Africa funders made up of the United States NIH and the Wellcome Trust) emphasise grants and contracts designed to support the initiation of a programme with a built-in sustainability plan for that reason. To address this, a fee-for-service model has been developed at I-HAB to support future biorepository activities. After projecting costs for labour, supplies, equipment maintenance and replacement, and overhead, a biorepository needs to add a mark-up to generate revenue for future investments in infrastructure upgrades and cost-of-operating increases. While academic and institutional-industrial partnerships are often considered to be a stable source of revenue, they are subject to the constraints of changing business goals. In addition, there are concerns about maintaining a strict ethical and logistical separation of biospecimens collected for ‘commercial’ programmes and those collected for ‘open collections’ accessible to the research community with ethical review and informed consent ensuring respect for patient confidentiality and privacy and a few other restrictions on the type of research. The most feasible approach to sustainability of an independent biorepository of quality will most likely involve a combination of the approaches that circumvent the challenges described above and that incorporate a strong and flexible business model.

### Conclusion

We have described the procedures, challenges and outcomes encountered in upgrading a study-specific sample storage facility to an internationally recognised biorepository supporting researchers globally with a focus on West Africa. During the process, key requirements for successful biobanking were identified: (1) institutional support for development of infrastructure and technical services, (2) scientific management staff on-site with primary commitment to the biorepository, (3) reliance on best practices from globally known biorepository groups, (4) early development and implementation of a QMS, (5) adoption of a LIMS with demonstrated versatility in user-defined fields and reporting functions and interoperability with other LIMS systems, (6) formal collaboration with external experts and sharing of experience through abstracts, newsletters, and published manuscripts, (7) strict adherence to local and national ethical guidelines, and (8) a sustainability plan that is reviewed and updated annually.

The I-HAB Biorepository at IHVN is now ideally positioned to utilise internationally recognised best practices to receive, process, store, and ethically redistribute high-quality biospecimens for research, innovation, discovery, and diagnostics for several years to come.

**Lessons learned**

- Despite infrastructural challenges and limited resources, it is possible to establish a biorepository in a resource-limited setting that operates at an international level, if resources are leveraged to support the methodical implementation of a strategy for improvement that is grounded in established best practices and continuous monitoring and adjustment to local challenges.
- Best practices, guidelines, standard operating procedures and training are the foundation for all procedures. However, it is also imperative to pilot all new procedures and processes prior to implementation to reveal obstacles that are unforeseeable, hidden or beyond the biorepository’s control.
- Stakeholders in a biorepository, including staff, interdisciplinary units, institutional administration, fellow biobankers, researchers, policymakers, ethical bodies, and commercial entities, must be fully engaged to communicate and actualise requirements, recommendations, and remediation in a reciprocal manner to achieve and maintain internationally accepted practices.

## References

[1] National Cancer Institute National Institutes of Health U.S. Department of Health and Human Services, NCI Best Practices for Biospecimen Resources Biorepositories and Biospecimen Research Branch, 2016.

[2] International Society for Biological and Environmental Repositories Best practices for repositories: Collection, storage, retrieval and distribution of biological materials for research International Society for biological and environmental repositories. 3rd edn. 2011, ISBER: Vancouver. 10.1089/bio.2012.1022 24844904

[3] Public Population Project in Genomics and Society (P³G). http://www.p3g.org/about-p3g. Documents section.

[4] H3Africa Consortium Enabling the genomic revolution in Africa: H3Africa is developing capacity for health-related genomics research in Africa. Science 2014;344(6190):1346–1348. 10.1126/science.1251546 24948725 PMC4138491

[5] ISBER Self assessment tool 2015. Available from: http://www.isber.org/?page=SAT.

[6] World Health Organization Regional Office for Africa 2012. Stepwise laboratory quality improvement process towards accreditation (SLIPTA) checklist version 2:2015 for clinical and public health laboratories. 2015;1–48. Available from: http://www.who.int/iris/handle/10665/204423.

[7] Integrated BioBank of Luxembourg Available from: https://www.ibbl.lu/ibbl-bioservices/biospecimen-proficiency-testing/.

[8] College of American Pathologists Accreditation Program in Biobanking 2017 Available from: http://www.cap.org/web/home/lab/accreditation/biorepository-accreditation-program?

[9] Osafo C, Raji YR, Burke D, et al Human Heredity and Health (H3) in Africa Kidney Disease Research Network: A focus on methods in Sub-Saharan Africa. Clin J Am Soc Nephrol. 2015;10(12):2279–2287. 10.2215/CJN.11951214 26138261 PMC4670777

[10] Akpalu A, Sarfo FS, Ovbiagele B, et al Phenotyping Stroke in Sub-Saharan Africa: Stroke Investigative Research and Education Network (SIREN) Phenomics protocol. Neuroepidemiology. 2015;45(2):73–82. 10.1159/000437372 26304844 PMC4604029

[11] Abimiku A, Croxton T, Akintunde E, et al Experiences in establishing a PEPFAR-supported laboratory quality system in Nigeria. Am J Clin Pathol. 2010;134(4):541–549. 10.1309/AJCP5RP4QWEQLUZR 20855634

[12] Abimiku A Building laboratory infrastructure to support scale-up of HIV/AIDS treatment, care, and prevention in-country experience. Am J Clin Pathol. 2009;131(6):875–886. 10.1309/AJCPELMG6GX6RQSM 19461097

[13] Nigerian Health Research Committee Available from: http://nhrec.net/.

[14] Rudan I, Marusic A, Campbell H, editors Developing biobanks in developing countries. J Global Health 2011;1(1):2–4. PMC3484738 23198094

[15] Staunton C, Moodley K Challenges in biobank governance in Sub-Saharan Africa. BMC Med Ethics 2013;14:35–42. 10.1186/1472-6939-14-35 24025667 PMC3849982

[16] Wonkam A, Mayosi B Genome genomic medicine in Africa: Promise, problems and prospects. Medicine 2014;6(2):11 10.1186/gm528 PMC3979013 25031612

[17] DeLuca A, Regenberg A, Sugarman J, et al Bioethical considerations in developing a biorepository for the pneumonia etiology research for child health project. Clin Infect Dis. 2012;54(Suppl 2):S172–S179. 10.1093/cid/cir1063 22403233 PMC3297549

[18] Agalliu I, Adebiyi A, Lounsbury D, et al The feasibility of epidemiological research on prostate cancer in African men in Ibadan, Nigeria. BMC Publ Health. 2015;15:425–436. 10.1186/s12889-015-1754-x PMC4419477 25927535

[19] Jespersen S, Tolstrup M, Hønge B, et al High level of HIV-1 drug resistance among patients with HIV-1 and HIV-1/2 dual infections in Guinea-Bissau. Virol J. 2015;12:41–46. 10.1186/s12985-015-0273-9 25889017 PMC4357169

[20] Ilboudo H, Noyes H, Julius Mulindwa J, et al Introducing the TrypanoGEN biobank: A valuable resource for the elimination of human African trypanosomiasis. PLoS Negl Trop Dis. 2017;11(6):e0005438 10.1371/journal.pntd.0005438 28570558 PMC5453417

[21] Abayomi A, Christoffels A, Grewal R, et al Challenges of biobanking in South Africa to facilitate indigenous research in an environment burdened with human immunodeficiency virus, tuberculosis, and emerging noncommunicable diseases. Biopreserv Biobank. 2013;11(6):347–354. 10.1089/bio.2013.0049 24835364 PMC4076990

[22] Roberts L, Goliath R, Rebello G, et al Inherited retinal disorders in South Africa and the clinical impact of evolving technologies. S Afr Med J. 2016;106(6 Suppl 1):S33–S37. 10.7196/SAMJ.2016.v106i6.10988 27245521

[23] Schneider JW, Sanderson M, Geiger D, et al A biobank to support HIV malignancy research for sub-Saharan Africa. S Afr Med J. 2016;106(9):867–869. 10.7196/SAMJ.2016.v106i9.10891 27601106 PMC5523654

[24] Barth DD, Engel ME, Whitelaw A, et al Rationale and design of the African group A streptococcal infection registry: The AFROStrep study. BMJ Open. 2016;6(2):e010248 10.1136/bmjopen-2015-010248 PMC4769387 26916694

[25] Nigeria H3Africa Website Available from: http://ihvnigeria.org/ihvnweb/webnew/index.php/officesdepts/clinical-lab-dept/bio-repository-unit.html.

[26] South Africa H3Africa Website Available from: http://www.cls.co.za/SPECIALIZED-FACILITIES.

[27] Uganda H3Africa Website Integrated Biorepository of H3Africa Uganda – IBRH3AU Available from: http://www.ibru.mak.ac.ug/.

[28] Mayne E, Croxton T, Abimiku, et al Genes for life: Biobanking for genetic research in Africa. Biopreserv Biobank. 2017;15(2):93–94. 10.1089/bio.2017.0007

[29] Abimiku A, Mayne E, Joloba M, et al Supporting genomics research on African populations by sharing high-quality biospecimens. Biopreserv Biobank. 2017;15(2):99–102. 10.1089/bio.2017.0005

[30] Croxton T, Swanepoel C, Musinguzi H, et al Lessons learned from biospecimen shipping among the Human Heredity and Health in Africa biorepositories. Biopreserv Biobank. 2017;15(2):103–110. 10.1089/bio.2017.0009

[31] Beiswanger C, Abimiku A, Carstens N, et al Accessing biospecimens from the H3Africa Consortium. Biopreserv Biobank. 2017;15(2):95–98. 10.1089/bio.2017.0008

[32] De Vries J, Tindana P, Littler K, Ramsay M, Rotimi C, Abayomi A, et al The H3Africa policy framework: Negotiating fairness in genomics. Trends Genet. 2015;31(3):117–119. 10.1016/j.tig.2014.11.004 25601285 PMC4471134

